# Assembly along lines in boundary-driven dynamical system

**DOI:** 10.1038/s41598-019-54160-8

**Published:** 2019-11-29

**Authors:** Kulveer Singh, Yitzhak Rabin

**Affiliations:** Department of Physics, and Institute of Nanotechnology and Advanced Materials, Bar-Ilan University, Ramat Gan, 52900 Israel

**Keywords:** Mathematics and computing, Physics

## Abstract

We introduce a simple dynamical rule in which each particle locates a particle that is farthest from it and moves towards it. Repeated application of this algorithm results in the formation of unusual dynamical patterns: during the process of assembly the system self-organizes into slices of low particle density separated by lines of increasingly high particle density along which most particles move. As the process proceeds, pairs of lines meet and merge with each other until a single line remains and particles move along it towards the zone of assembly. We show that this pattern is governed by particles (attractors) situated on the instantaneous outer boundary of the system and that both in two and in three dimensions the lines are formed by zigzag motion of a particle towards a pair of nearly equidistant attractors. This novel line-dominated assembly is very different from the local assembly in which particles that move towards their nearest neighbors produce point-like clusters that coalesce into new point-like clusters, etc.

## Introduction

Systems composed of a large number of autonomous agents interacting with simple rules often exhibit emergent large scale behavior^1^. In living systems such as a bacterial swarms^2–4^, flocks of birds^5,6^, schools of fish^7,8^, etc.^9,10^, a highly coordinated movement among the agents arises in the absence of centralized control, due to the action of individual agents based on the perception of their local environment (i.e the behavior of each agent is determined by that of its neighbors). These synchronized motions observed in natural systems were successfully modeled using algorithms based on local behavioral rules of autonomous agents in computer simulations^11–14^. For example, using three simple rules, viz. collision avoidance, velocity matching, and flock centering in his ‘BOID’ model^15^, Craig Reynolds has simulated a coordinated group movement in a flock of birds.

In this paper we propose and analyze a simple non-local algorithm for aggregation of agents according to which, at each moment, every agent senses the locations of all the other agents and moves towards of the farthest agent from it. This rule implies that the behavior of each agent in a swarm is determined only by agents located at the outer boundary of the swarm. Continued application of this simple rule results in the appearance of anisotropic dynamical patterns composed of low density “slices” separated by high density lines. As the system contracts, agents in the slices migrate towards the lines that separate between neighboring slices and continue to move along them towards a gathering point whose position is close to but not coincident with the center of mass of the initial system. In the course of contraction, the number of the slices and of the associated lines decreases due to their coalescence. We show that the dynamic patterns produced by this simple non-local algorithm are qualitatively different from those that arise using a local rule, where an agent moves towards its nearest neighbor.

## Model

Consider a swarm of *N* autonomous agents initially randomly distributed in a region bound by a circle (in 2d). Each agent is modeled as a point particle whose position is updated according to the following simple rule: For each particle (*i*) find the particle (*j*) that is furthest from it at this time and if there are several particles whose distance from particle *i* is the same, randomly choose one of them (while possible in principle, such exact degeneracy was never observed in our simulations). Next, move all particles simultaneously by distance Δ*x* towards their furthest particles.

The above steps are performed by every particle in each iteration (time-step) and therefore, the choice of the farthest particle may change with time. According to this algorithm, the interaction between the particles is not always reciprocal, in the sense that if particle *j* is farthest from particle *i*, it is not necessarily the case that *i* is farthest from *j*. Also, since each particle is affected only by the one particle that is farthest from it and is therefore located at the outer periphery of the system, every particle will move towards the far boundary. The combined effect of such displacements of all the particles (including the boundary particles themselves) towards the far boundary, results in the contraction of the system and the assembly of all the particles in it. The time *t* is equal to number of discrete time-steps starting from *t* = 0.

## Results and Discussion

**Figure 1:**
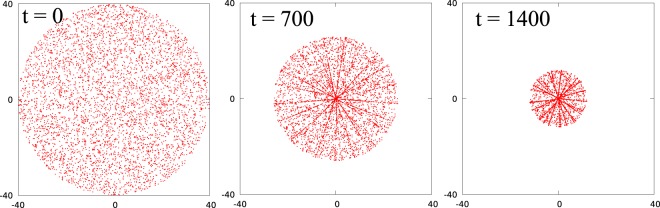
Snapshots of a system of 5000 particles at three different times. At *t* = 0, particles are randomly distributed inside a circular region. Particles form lines in the system (*t* = 700) and density of particles on the lines increases as time progresses (*t* = 1400).

**Figure 2:**
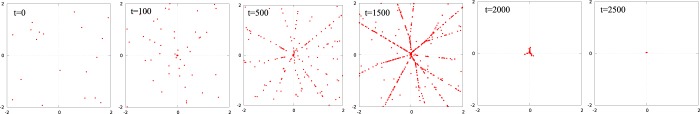
Snapshots of the system near the assembly zone at six different times (expanded view). The position of the assembly zone is determined at an early stage of evolution (*t* = 100). Several lines meet at the assembly zone; the other lines appear to branch out of these lines (*t* = 1500). Density of particles on the lines increases as time progresses (see *t* = 500, 1500). Towards the end of the process, the number of lines decreases to three (*t* = 2000) and finally all the particles accumulate in the assembly zone (*t* = 2500).

We began the simulation by randomly placing *N* = 5000 particles inside a circle of radius $R=\sqrt{N/(\pi \rho )}$ with uniform surface density *ρ* = 1/*σ*^2^ and chose *σ* as the unit to measure distances (*σ* = 1), and the displacement step Δ*x* = 0.02. Figure 1 shows snapshots of the system at three different times (also see Movie M1 in SI). As evident from the snapshots, while initially (at *t* = 0) the distribution of particles is uniform and isotropic, the distribution becomes anisotropic as the system evolves and the rotational symmetry is spontaneously broken. Thus, as particles move inward and the system contracts, it self-organizes into slices of low particle density separated by lines of high density of particles. The formation of lines begins quite early and the density of particles within the lines increases with time. Note that the motion of neighbouring particles along each line is strongly correlated despite the fact that our algorithm does not allow the particles to sense their local environment. The radius of the particle distribution shrinks and all the particles move towards the centre of the circle, as time progresses. Eventually, all the particles assemble in a region of width 2Δ*x* near the center of the circle, defined as the assembly zone. Figure 2 shows snapshots of a small section of the system around the assembly zone (see Movie M2 in SI). As the system evolves, particles begin accumulating in the assembly zone which remains almost fixed till the end of the process. We also observe that only very few lines merge directly at the assembly zone, with the other lines branching out of these lines. The total number of lines in the system decreases as the particle distribution shrinks. Towards the end of the assembly process the number of lines decreases to three and then to one, and finally all the particles accumulate in the assembly zone (see Fig. 2). Furthermore, every particle enters the assembly zone along these lines only (see Movie M2 in SI). The particles in the low density slices between the lines, move towards these lines and join them. In order to make sure that the above picture of the dynamics is robust, we repeated the simulations with many different initial conditions and did not find any qualitative differences in the assembly process.

To confirm that the formation of lines is not limited to circular geometry, we performed simulations of random particle distributions in other initial geometries i.e., square and semi-circular domains, and observed four lines in square geometry and only one line (initially) in semi-circular geometry in all the simulations (see Movies M3 and M4 in SI). Contrary to these systems, the initial number of lines in the circular geometry was observed to depend, albeit weakly, on the initial conditions. This makes the circular geometry case more interesting and complex as compared to other geometries. We also checked the dynamics for larger displacements Δ*x* = 2 and 5 (i.e., larger than the mean initial interparticle distance *σ* = 1) and found that the number of lines does not depend on the choice of the step size but that the width of these lines increased with increasing Δ*x*. In the remainder of the paper, we systematically explore the mechanisms behind the formation of the dynamical patterns observed in the circular geometry.

**Figure 3:**
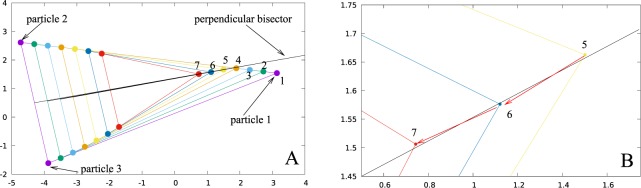
(**A**) Three particle system: differently colored vertices of the triangles represent the positions of the particles at different instants of time. The positions of particle 1 at various times are labeled as 1, 2,...., 7. (**B**) Expanded view of the trajectory of particle 1 which executes a zigzag motion along the perpendicular bisector to the line between particles 2 and 3 as it comes within Δ*x* from the bisector (see positions 5, 6, 7 of particle 1).

In order to understand the formation of the lines, we turn to examine the dynamics of a smaller system. Since the two particle case is trivial as both particles simply move towards each other along the straight line joining them, we consider a three particle system. At any instant the positions of the three particles can be thought of as the vertices of a triangle, and in our algorithm the lengths of the sides of this triangle determine the direction of motion of the particles. The two particles which form the longest side of the triangle move towards each other and the third particle moves along the second longest side. In Fig. 3A, we plot the positions of the three particles at different times (different colors represent different time instants). We observe that starting from any triangle, the system reaches a stage where three particles form a quasi-isosceles triangle in which two of the longer sides have nearly equal lengths (the lengths differ by less than the step size Δ*x*). Since at time 1, particle 1 is the farthest from both particles 2 and 3, it acts as an attractor for these particles and they move towards it. Turning our attention to the motion of particle 1, we observe at time instants 1, 2 and 3 in Fig. 3A, particle 1 moves towards particle 2 until it comes within Δ*x* of the perpendicular bisector of the line joining particles 2 and 3, and forms a quasi-isosceles triangle with these particles (see Movie M5 in SI). Once particle 1 enters this region, the difference between the distances of other two particles from it becomes of the order of step size (Δ*x*) at which point it has two nearly equidistant attractors. In the next one or two steps, particle 1 crosses the perpendicular bisector and the farthest particle from it becomes particle 3. Now particle 1 moves towards particle 3 and again crosses the perpendicular bisector at which time the farthest particle from it again becomes particle 2. This frequent switching between the two attractors continues and leads to zigzag motion of particle 1 about the perpendicular bisector (see Fig. 3B). Since the amplitude of the zigzag motion is of the order of step size which we have chosen to be very small (Δ*x* = 0.02) compared to the average interparticle distance, particle 1 appears to move along a straight line which is the perpendicular bisector of the side joining the two nearly equidistant attractors, particles 2 and 3.

Having understood that zigzag motion of a particle along the bisector of two neighboring nearly equidistant attractors in a three particle system appears (at sufficiently low resolution) as motion along a line, we return to the large system case (5000 particles) in the circular geometry. A careful examination of the dynamics of assembly shows that while particles on the lines execute zigzag motion (changing direction abruptly and very frequently), all particles inside a “slice” located between neighboring lines move smoothly towards a common point of convergence (see Movie M6 in SI where velocity vectors of particles evolving with time are shown). This concurs with the expectation that while particles in the interior of a slice move towards a common attractor located near the far outer boundary of the system, those on the boundary line between two neighboring slices execute a zigzag motion whose direction alternates between one of the two nearly equidistant attractors and therefore oscillates around the bisector to the line connecting these attractors. The fact that many particles move along the same line indicates that these particles have a common nearly equidistant pair of attractors. The number of lines is identical to the number of attractors in the system. The particles in the slices between the lines move closer to the lines during the process of contraction and eventually join these lines before entering the assembly zone.

**Figure 4:**
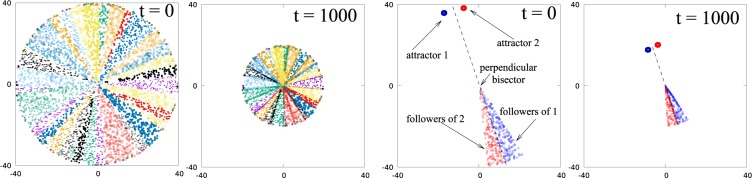
Followers of each attractor (towards which they move), are represented by points of the same type and same color. With this convention the system splits into the slices of different colors where each slice corresponds to a different attractor. In the right panel we show two neighboring slices and their corresponding attractors. The boundary between two slices defines a line that lies on the perpendicular bisector of two neighboring attractors.

According to our dynamical rules, at any instant of time each particle in the system moves towards another particle (its attractor) and therefore can be termed as the follower of this attractor. Note that all the attractors are located in a narrow annular region close to the outer boundary of the system at time *t* and therefore the total number of attractors *N*_*A*_(*t*) is much smaller than the number of particles in the system *N*. Since each attractor is a follower of some other attractor, the total number of followers is *N*. In order to visualize the way in which the system separates into groups of followers of different attractors, the followers of different attractors are shown in different colors in Fig. 4. This scheme divides the system into differently colored slices where each slice contains the followers of one attractor (see Movie M7 in SI). Thus, the number of such slices is equal to the number of attractors and the area of each slice is proportional to the number of followers of the corresponding attractor. The border line between neighbouring slices is formed by the perpendicular bisector of the line joining their attractors (see right panel in Fig. 4 where only two neighbouring slices are shown).

**Figure 5:**
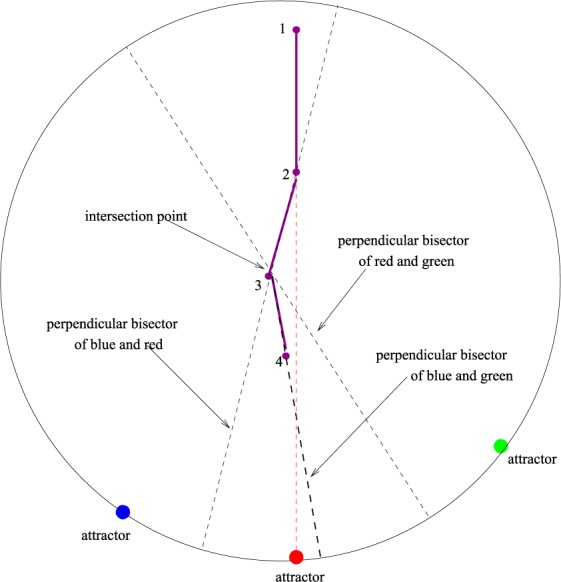
Schematic view of the dynamics of a typical follower of the middle (red) attractor.

Inspection of Movie M7 in the SI shows that the number of attractors and that of the corresponding slices of followers and of the associated border lines, decreases with time. The way it happens is demonstrated in Movie M8 in SI where we show three neighboring blue, red and green attractors and their corresponding slices of followers and observe that the area of the middle slice decreases with time and eventually disappears completely, together with its attractor. In order to understand the mechanism behind the decrease of *N*_*A*_(*t*) with time, in Fig. 5 we present a simplified picture of the above process in which we freeze the attractors and schematically describe the motion of a particle in the slice that corresponds to the middle (red) attractor. As shown in the figure, as the particle moves from point 1 towards its attractor, it reaches the boundary line which is the perpendicular bisector of the imaginary line between the blue and the red attractors (point 2). From this time on it continues to move (in a zigzag fashion) along this bisector until it approaches the intersection point (point 3) of two neighbouring perpendicular bisectors which is the circumcenter of the triangle formed by the corresponding three attractors and is therefore equidistant from the three of them. This threefold degeneracy is removed as the particle moves away from the intersection point by a small amount ~Δ*x* and the distance between the particle and the red attractor becomes smaller than the distance to the blue and the green attractors. From this time on (point 4), the red point on the boundary stops being an attractor for the particle and the blue and green points become its quasi-degenerate pair of attractors. The two lines formed by the two neighboring pairs merge to form another line which is the perpendicular bisector of the imaginary line joining the blue and green attractors. Similar dynamics takes place for all the particles in the central slice in Fig. 5, leading eventually to the disappearance of the red attractor.

**Figure 6:**
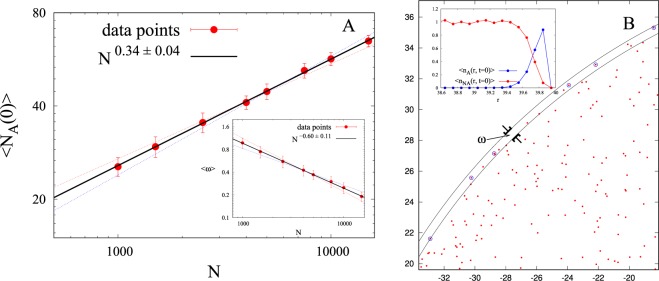
(**A**) Plot shows that 〈*N*_*A*_(0)〉 scales as *N*^0.34±0.04^, where *N* is the total number of particles in the system. Inset to the figure shows that the averaged (over all realizations) width of the smallest annulus which accommodates all the attractors at *t* = 0 scales as *N*^−0.60±0.11^ (**B**) Snapshot shows the annulus and the particles in it (red dots) within the zoomed portion of a circle. Particles which are attractors are circled in blue. Inset of (**B**) shows the number of attractors per unit area (〈*n*_*A*_(*r*, *t* = 0)〉) and number of non-attractors per unit area (〈*n*_*NA*_(*r*, *t* = 0)〉) as a function of distance from the centre of the circle.

To quantify the results we carried out simulations for eight different densities in the range *ρ* = 0.2−3.0 (in units of *σ*^−2^), keeping the radius *R* of the circle fixed in all the simulation runs. For each density we ran the simulation for 50 different initial realizations (different random choices of particle positions within the circular region) and computed the average number of attractors (〈*N*_*A*_(0)〉) at time *t* = 0 in the system. Figure 6A shows the dependence of 〈*N*_*A*_(0)〉 on the total number of particles *N* = *ρπR*^2^ in the system (log-log plot). From the slope of the line we find that 〈*N*_*A*_(0)〉 ∝ *N*^0.34±0.04^. We then computed the width of the annulus (*ω*) along the periphery where all the attractors lie and found that 〈*ω*〉 ∝ *N*^−0.6±0.11^ (see inset of Fig. 6A). Figure 6B shows a snapshot of particles (red dots) inside and outside the annulus of width *ω* of a part of the circular region. Particles which are attractors are circled with blue color. Clearly, not all the particles inside the annulus are attractors. We computed the average number of attractors per unit area 〈*n*_*A*_(*r*, *t* = 0)〉 and average number of non-attractors per unit area 〈*n*_*NA*_(*r*, *t* = 0)〉 (averaged over angles and over different realizations) as a function of distance from the center (see inset of Fig. 6B). Note that the radial density of attractors 〈*n*_*A*_(*r*, *t* = 0)〉 gradually increases whereas that of non-attractors 〈*n*_*NA*_(*r*, *t* = 0)〉 decreases as one goes from the inner to the outer periphery of the annulus. The average number of particles (〈*N*_*annulus*_(0)〉 = 〈*N*_*A*_(0) + *N*_*NA*_(0)〉) within the annulus can be written as 〈*N*_*annulus*_(0)〉 ≈ 2*πR*〈*ω*〉*ρ*. Since R is constant for all densities, we can write 〈*N*_*annulus*_(0)〉 ∝ 〈*ω*〉*ρ* where *ρ* ∝ *N*. From simulations we find that 〈*ω*〉 ∝ *N*^*β*^, where *β* = −0.6±0.11 and therefore 〈*N*_*annulus*_〉 ∝ *N*^1 + *β*^ = *N*^0.4±0.11^. Comparing 〈*N*_*A*_(0)〉 and 〈*N*_*annulus*_(0)〉 we conclude that both have approximately the same scaling exponent with *N* (within error bars). This is confirmed by directly computing 〈*N*_*annulus*_(0)〉 in the simulations (see Fig. S1 in SI) which yield 〈*N*_*annulus*_(0)〉:〈*N*_*A*_(0)〉 ≈ 2:1. We also examined the case where density of all the systems is constant and number of particles is varied by changing the radius of the circular region and obtained the same scaling of the number of attractors with *N* (see Fig. S2 in SI).

**Figure 7:**
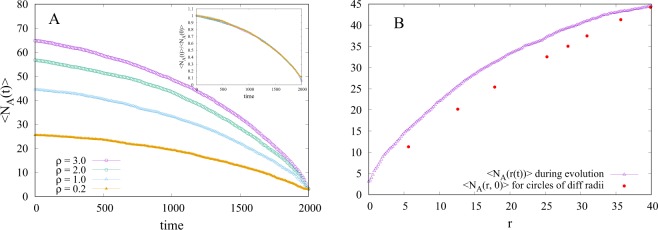
(**A**) 〈*N*_*A*_(*t*)〉 vs *t* plot for circular geometry of same radius and four different densities (*ρ* = 0.2, 1.0, 2.0, 3.0). 〈*N*_*A*_(*t*)〉/〈*N*_*A*_(0)〉 is plotted in the inset where data of all four densities collapses to give universal decay behavior of relative number of average attractors. (**B**) Comparison of 〈*N*_*A*_(0)〉 computed for different radii and *ρ* = 1 assuming that particles are uniformly distributed, with 〈*N*_*A*_(*r*)〉 obtained when the radial distribution shrinks during dynamic evolution.

In Fig. 7A we plot the average (over initial realizations) number of attractors, 〈*N*_*A*_(*t*)〉 in the system as a function of time, *t*, for different particle densities. In the inset of Fig. 7A, we collapse the different curves on a single universal plot for different values of the density, by normalizing 〈*N*_*A*_(*t*)〉 by 〈*N*_*A*_(0)〉. Therefore,

1 $$\frac{\langle {N}_{A}(t)\rangle }{\langle {N}_{A}\mathrm{(0)}\rangle }=f(t),$$

where *f*(*t*) is a universal function of time that does not depend on particle density. Interestingly, *f*(*t*) can not be fitted by a simple/stretched exponential or by power law decay and we were not able to come up with an analytic model for it. Next, from the initial distribution of particles, we computed the average number of attractors 〈*N*_*A*_(*r*, 0)〉 for circles of different radii (r) and the same particle density. We performed simulations for three different densities *ρ* = 0.5, 1.0, 2.0 and found that scaling 〈*N*_*A*_(*r*, 0)〉 by 〈*N*_*A*_(*R*, 0)〉, where *R* is the radius of largest circle considered at each density, collapses data of all three densities on top of each other, thus giving a universal curve (see Fig. S3 in SI). To compare it with 〈*N*_*A*_(*t*)〉 obtained when the particle distribution shrinks during dynamic evolution, we computed the radius *r* as a function of *t* and found that *r* = −0.02*t* + 40 (see Fig. S4 in SI). Using this result we converted the x axis of Fig. 7A from time (t) to radius (r). Figure 7B shows the comparison of 〈*N*_*A*_(*r*, 0)〉 with the average number of attractors obtained at different radii 〈*N*_*A*_(*r*(*t*))〉 during dynamic evolution (for *ρ*  = 1). 〈*N*_*A*_(*r*(*t*))〉 is larger than 〈*N*_*A*_(*r*, 0)〉 at a particular radius of the circle (see Fig. 7B), presumably because the system does not remain uniform and radial density increases with decreasing r(t) (see Fig. S4 in SI), as the system evolves. We conclude that both the shrinking of the particle distribution and the change of the density profile during the dynamic evolution (collapse) of the system play an important role in the decay of the number of attractors with time.

**Figure 8:**
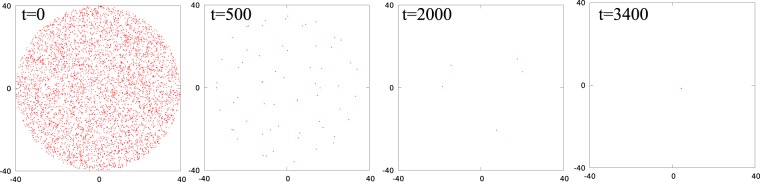
Snapshots of a system of 5000 particles at four different times where particles move towards their nearest neighbour. At *t* = 0, particles are randomly distributed inside a circular region. Particles coalesce to form groups (*t* = 500), these groups coalesce again to form groups containing large numbers of particles (*t* = 2000) and the process continues till all particles assemble into a single cluster (*t* = 3400).

The non-local dynamical rule we proposed can be used to assemble all the agents at a certain location in space and it is instructive to compare its efficiency to that of a local algorithm according to which at every time step each particle finds the closest particle and moves towards it. In order to avoid short distance singularities, we introduce the constraint that if the separation between the particles is smaller than Δ*x* = 0.02, they stop sensing each other and each of them moves towards the next nearest particle located at distance larger than 0.02. Repeated application of this algorithm results in the formation of numerous point-like (of size < Δ*x*) clusters of particles. These clusters coalesce to form new point-like clusters composed of increasingly larger numbers of particles, a process reminiscent of nucleation and growth in phase separating systems^16^. This process continues until all *N* particles assemble into a single point-like cluster of size < Δ*x*. Figure 8 shows the snapshots, at different times, of a system of 5000 particles (initially randomly placed in a circular region with density *ρ* = 1/*σ*^2^) evolved using this algorithm (also see Movie M9 in SI). We computed the total time of assembly of all the particles using this algorithm and compared it with the collapse time of our non-local algorithm. We found that the system assembles much faster using the non-local (*t* = 2200) than the local (*t* = 3400) algorithm. Another point to notice is that the assembly zone is determined very early by the system using the proposed non-local algorithm and the particles always move towards the assembly zone. Particles which are closer to the assembly zone move over a small distance, while those that are further away from this zone have to move over large distances to reach their final destination. Conversely, the assembly zone is determined very late in systems using a local algorithm and particles do not always move towards the assembly zone. Consequently, most particles cover large distances before reaching the assembly zone.

In order to check whether and how the assembly of particles depends on the dimensionality of the system, we performed simulations using the non-local algorithm in one and in three dimensions. In the 1D case, we randomly placed *N* = 100 particles on a line between −*L* to *L*, where 2*L* = *N*/*ρ*_1*d*_ with uniform linear density *ρ*_1*d*_  = 1/*σ*. In this case the motion of all the particles is determined only by the two boundary particles that are the closest to ends of the line, i.e. to −*L* and *L*. All particles which are on one side of the mid-point M of the line joining these two boundary particles, move along this line towards the particle closest to end of the line on the other side of M. The two boundary particles remain the attractors of the system until the end of the collapse when all the particles reach the assembly zone around M (see Movie M10 in SI). In 3D, we randomly placed *N* = 10000 particles in a spherical region of radius *R* = (3*N*/4*πρ*_3*d*_)^1/3^ with uniform density *ρ*_3*d*_ = 1/*σ*^3^. Similar to the 2D case, we observed that the collapse proceeds through formation of lines that radiate outward from a point close to the centre of the sphere (see Fig. S5 in the SI). Although visualization is more difficult in 3D than in 2D, we conclude that each of the lines is the common tangent to neighboring cones each of which contains the followers of a given attractor (not shown), that replace the slices shown in Fig. 4.

## Conclusions

In this paper, we simulated an ensemble of particles randomly distributed in a circular region in two dimensions that follow a simple dynamical rule: every particle (follower) moves towards the farthest particle (attractor) from it. An obvious consequence of this dynamical rule is that the attractors are always located near the instantaneous outer boundary of the system and constitute a small fraction of the total number of particles *N*. As a follower moves towards its attractor, it approaches the perpendicular bisector of the imaginary line joining this attractor to its neighbouring attractor, and from this point on it executes a zigzag motion about this bisector as it switches between the two attractors; since deviations from the line are small, it appears that the particle moves along the line. As time progresses, the system collapses but this collapse is anisotropic: the initially isotropic system self-organizes into slices of low particle density that are separated by lines of increasingly higher density and eventually most particles move along these lines towards the assembly zone. We find that the initial number of attractors 〈*N*_*A*_(0)〉 scales as *N*^0.34±0.4^ and decreases with time as some of the attractors lose their followers and therefore forego their status of attractors; plotting the ratio 〈*NA*(*t*)〉/〈*N*_A_(0)〉 vs *t* yields a universal curve for all densities. We also found that line formation is not limited to circular geometry: lines are observed in square and semi-circular geometries as well, even though the number of lines in these geometries is much smaller and does not strongly depend on initial conditions. Formation of lines in a circular geometry was observed for random non-uniformly distributed 2D particle systems as well, e.g. for radially non-uniform distribution (density varying as 1/*r*) and hyper-uniform distribution^17^ and also in a non-random system in which particles were placed on a square lattice bounded by a circle. We found that collapse along lines is a unique feature of our non-local dynamical rule and takes place in in 1, 2 and 3 dimensions. Even though the observation of such a collapse in a 1D system appears to be trivial, it is actually not. For example, if one uses a local rule according to which particles move towards their nearest neighbors, the dynamics leads to the formation of many point-like clusters (each composed of several particles) which continue to coalesce until a single point-like cluster that contains all the particles in the system remains. This should be contrasted with the 1D dynamics produced by the non-local rule where all the particles move uniformly towards the center of the 1D distribution. Interestingly, the time of assembly of a system evolved using the non-local rule is shorter than that for the local rule. While the non-local rule appears to be unphysical for most natural systems in which interactions are local in character, it can be implemented in artificial agent systems e.g., robots that can communicate across arbitrary distances^18–24^.

## Supplementary information

Supplementary information

Supplementary information

Supplementary information

Supplementary information

Supplementary information

Supplementary information

Supplementary information

Supplementary information

Supplementary information

Supplementary information

Supplementary information
