# Supplementary material for: Assembly along lines in boundary-driven dynamical system
Source: Sci Rep. 2019 Nov 29;9:17910. doi: 10.1038/s41598-019-54160-8 (PMC6884450; doi:10.1038/s41598-019-54160-8)
Supplement: Supplementary file 1 — Supplementary information [file 41598_2019_54160_MOESM1_ESM.pdf]

Supplementary Material: Assembly along lines in boundary-driven dynamical system

Kulveer Singh[1,*] and Yitzhak Rabin[1,†]

[1]*Department of Physics, and Institute of Nanotechnology and Advanced Materials, Bar-Ilan University, Ramat Gan 52900, Israel*

(Dated: November 1, 2019)

I. MOVIES

1. M1: Assembly process of particles in circular region.
2. M2: Assembly process of particles in circular region near assembly zone.
3. M3: Assembly process of particles in square geometry.
4. M4: Assembly process of particles in semi-circular geometry.
5. M5: Three particle system assembly process.
6. M6: Assembly process of particles in circular region. Velocity vectors of each particles are plotted.
7. M7: Assembly process of particles in circular region. Followers of one attractors are colored with same color. System splits into different color slices.
8. M8: Evolution of three neighbouring slices. Movie shows the process of decrease in number of attractors.
9. M9: Assembly process of particles in circular region via local interacting algorithm.
10. M10: Assembly process of particles in 1D system.

[*]Electronic address: kulveersingh85@gmail.com

[†]Electronic address: yitzhak.rabin@biu.ac.il

II. S1: SCALING OF $\langle N_{annulus}(0)\rangle$ AND $\langle N_A(0)\rangle$ WITH N

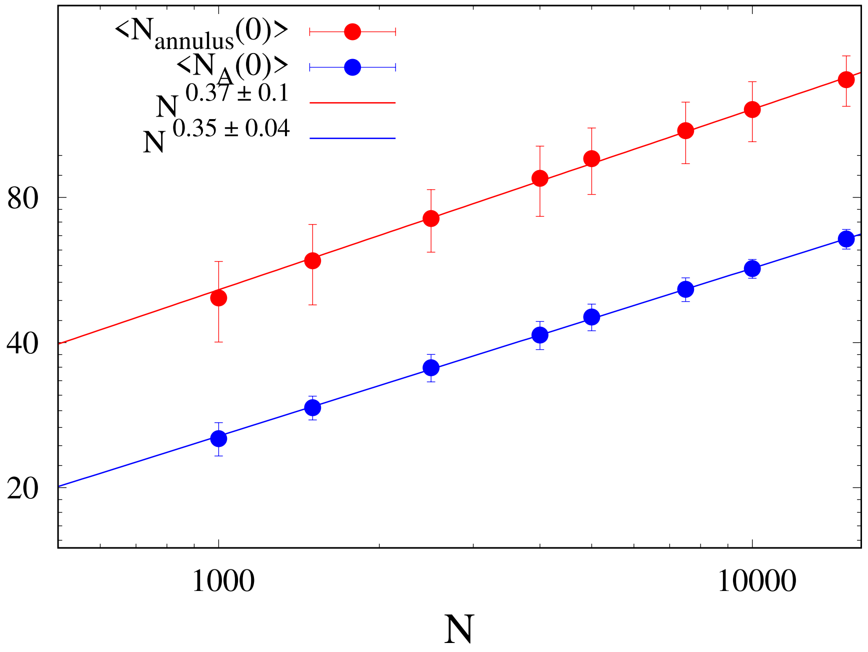

FIG. S 1: Plot shows $\langle N_{annulus}(0)\rangle$(total number of particles in annular region) and $\langle N_A(0)\rangle$ as a function of total number of particles (N) for different densities. Scaling exponent is roughly the same (within error bars) for both $\langle N_{annulus}(0)\rangle$ and $\langle N_A(0)\rangle$ and $\langle N_{annulus}(0)\rangle/\langle N_A(0)\rangle \approx 2$.

III. S2: SCALING OF $\langle N_A(0)\rangle$ WITH N FOR CONSTANT DENSITY CASE

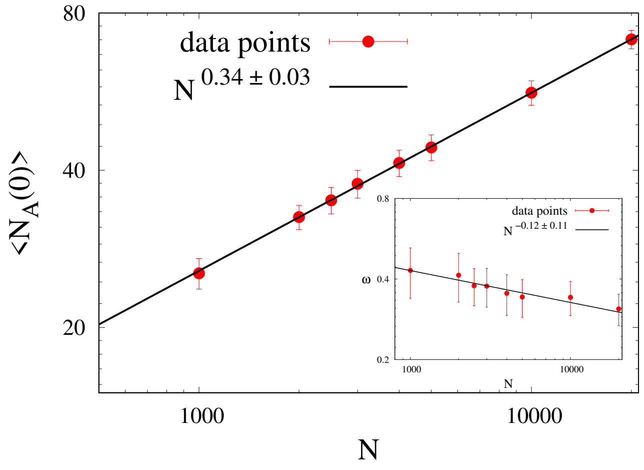

FIG. S 2: Plot shows $\langle N_A(0)\rangle$ as a function of total number of particles (N) for $\rho = 1$. The mumber of particles in the system is varied by changing the radius of the disc. $\langle N_A(0)\rangle$ scales as $N^{0.34\pm0.03}$ whereas the $\langle\omega\rangle$ scales as $N^{-0.12\pm0.1}$ (recall that $\langle\omega\rangle$ scales as $N^{-0.6\pm0.11}$ for the fixed case discussed in the manuscript). For constant density case ,we can write $\langle N_{annulus}(0)\rangle \approx 2\pi R\langle\omega\rangle\rho \Rightarrow \langle N_{annular}(0)\rangle \propto R\langle\omega\rangle$. Since $R \propto N^{1/2}$ and $\langle\omega\rangle \propto N^{\beta}$, where $\beta = -0.12 \pm 0.1$, we get $\langle N_{annular}(0)\rangle \propto N^{1/2+\beta} = N^{0.38\pm0.1}$, i.e. the scaling exponent is approximately the same (within error bars) as that of $\langle N_A(0)\rangle$.

IV. S3: $\langle N_A(r,0)\rangle$ VS RADIUS FOR DIFFERENT DENSITIES

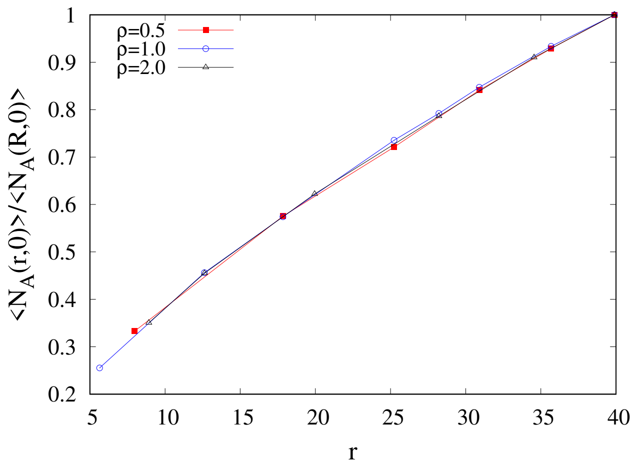

FIG. S 3: $\langle N_A(r)\rangle$ are computed by uniformly distributing the particles in circular regions of different radii keeping the density constant. Simulations are performed for three different densities. Plot shows $\langle N_A(r)\rangle/\langle N_A(R)\rangle$ vs r, where R is the radius of the largest disc considered for each density. Scaling $\langle N_A(r)\rangle$ by $\langle N_A(R)\rangle$ collapses the data of three densities on the same universal curve.

V. S4: RADIUS OF DISK AND RADIAL DENSITY DISTRIBUTION AS A FUNCTION OF TIME

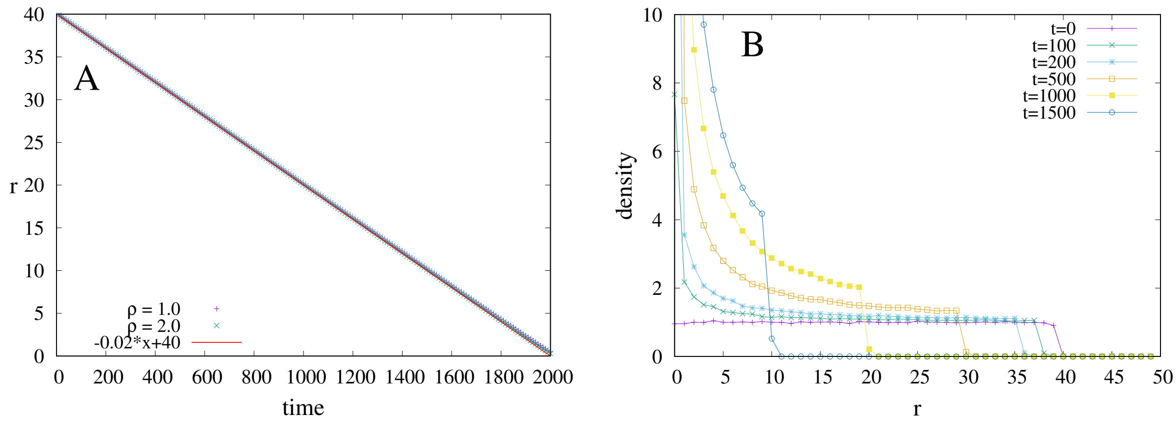

FIG. S 4: (A) Plot of the radius of disc as a function of time for $\rho = 1, 2$. Radius decreases linearly with time and is given by $r \approx -0.02t + 40$. (B) The time evolution of radial density distribution for $\rho = 1$.

VI. S5: ASSEMBLY ALONG LINE IN 3D SYSTEM

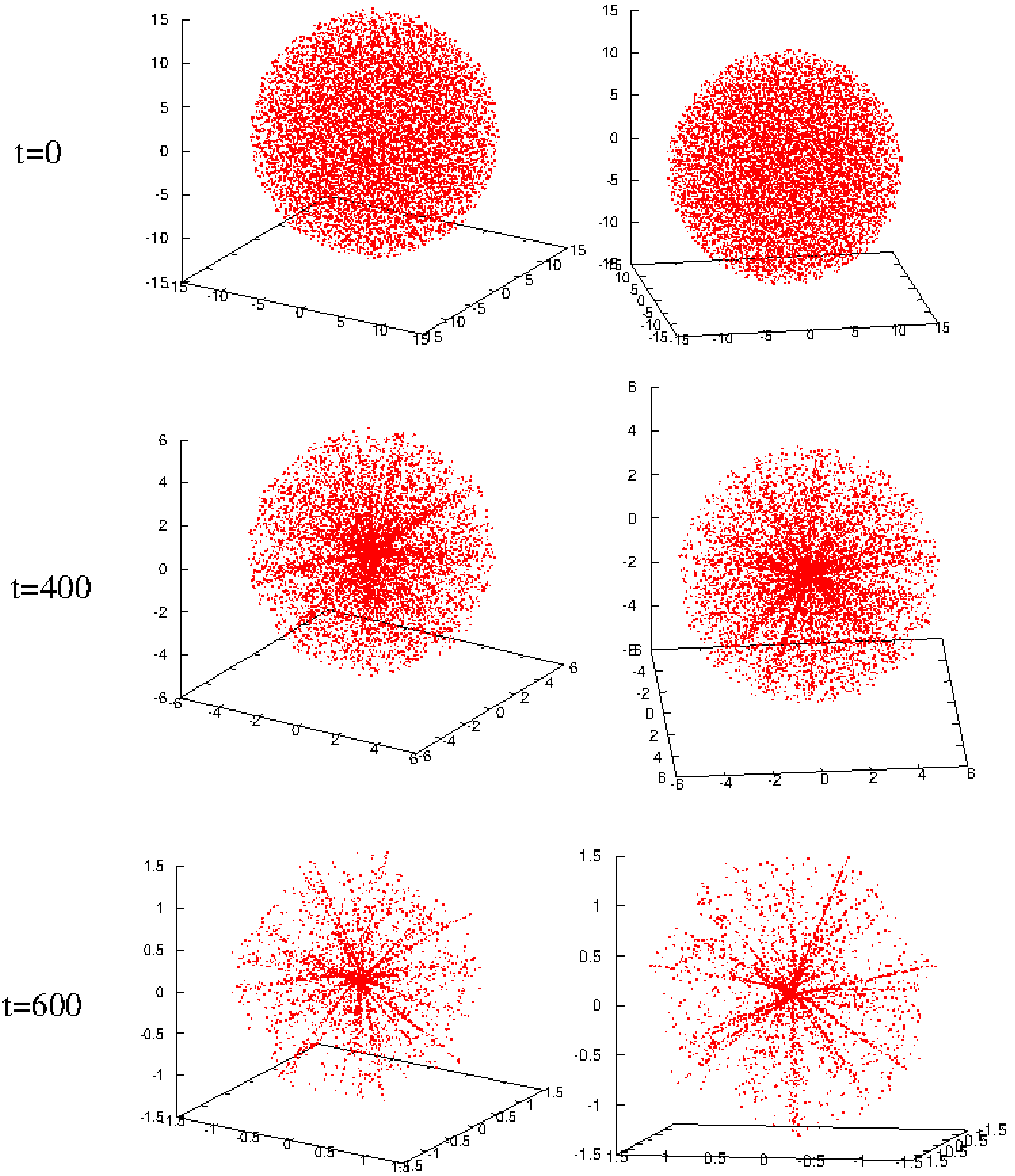

FIG. S 5: Snapshots of collpase of 3D system at three different times. Two snapshots from different angle view for each time instant are shown. Particles are initially($t = 0$) distributed uniformly inside the sphere. Assembly occurs via formation of lines which are visible at later times ($t = 400, 600$).
